# Non-fluoroscopic Cardioneuroablation for Deglutition-induced Syncope: Not a Bitter Pill to Swallow

**DOI:** 10.19102/icrm.2023.14037

**Published:** 2023-03-15

**Authors:** Andrés F. Miranda-Arboleda, Cengiz Burak, Hoshiar Abdollah, Adrian Baranchuk, Tolga Aksu, Andres Enriquez

**Affiliations:** ^1^Queen’s University, Kingston Health Science Centre, Kingston, Ontario, Canada; ^2^Department of Cardiology, Yeditepe University, Faculty of Medicine, Istanbul, Turkey

**Keywords:** Cardioneuroablation, syncope, zero fluoroscopy

## Abstract

Swallowing is an uncommon trigger of reflex situational syncope. We discuss the case of a 61-year-old woman who presented without a prior cardiac history complaining of 15 years of dizzy spells and hot facial flushing provoked by the swallowing of solid foods.

## Introduction

Swallowing is an uncommon trigger of reflex situational syncope.^1^ Its pathophysiology is probably related to the stimulation of neurological pathways that cause vagal hyperactivation, generating bradyarrhythmias, hypotension, and syncope.^2^

Lifestyle modifications and permanent pacemaker (PPM) implantation are the cornerstones of swallow syncope treatment; however, alternative interventional therapies have emerged during the last few years.^1,3^ A patient with a longstanding history of swallowing-induced syncope who was successfully treated with cardioneuroablation (CNA) as an alternative to PPM is discussed.

## Case presentation

**Figure 1:**
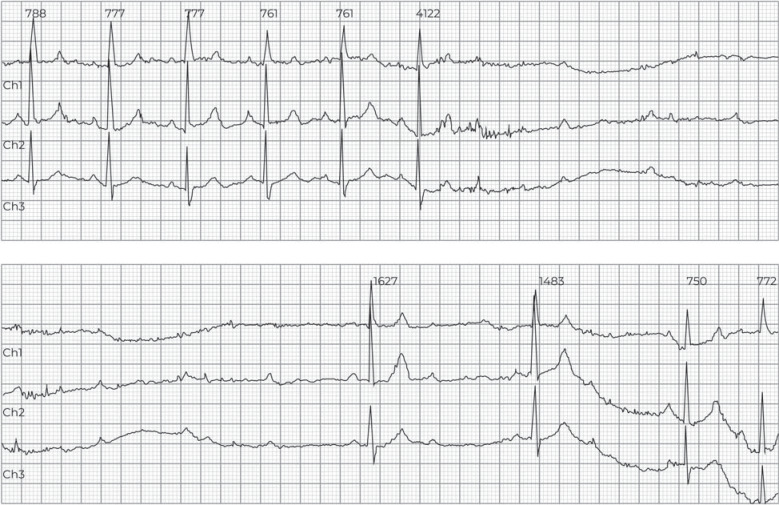
Holter monitor rhythm strips showing episodes of high-degree atrioventricular block while the patient was eating.

A 61-year-old woman without a prior cardiac history presented, complaining of 15 years of dizzy spells and hot facial flushing provoked by the swallowing of solid foods. Her symptoms were initially attributed to menopause; however, they did not subside over time, and she started experiencing recurrent syncope. A 72-h Holter monitor evidenced multiple episodes of high-degree atrioventricular (AV) block **(Figure 1)** a without significant change in the sinus rate or P–R prolongation preceding the AV block. All these episodes occurred while she was eating.

**Figure 2:**
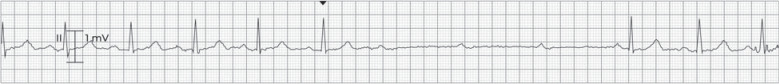
Pre-ablation swallowing tests were performed while eating a sandwich.

She was admitted to hospital for a cardiovascular workup and consideration of a pacemaker implant. Her echocardiogram showed no evidence of structural heart disease, and cardiac magnetic resonance imaging did not suggest infiltrative disease. Computed tomography of the head and neck and gastrointestinal endoscopic studies showed no significant findings. While in the hospital, her symptoms and AV block were easily reproducible by ingestion of a wholegrain cheese sandwich **(Figure 2)**.

Implantation of a dual-chamber pacemaker was considered the first therapeutic option; however, CNA was discussed as an off-label alternative. The patient was not keen to proceed with permanent pacing, and she opted for ablation.

**Figure 3:**
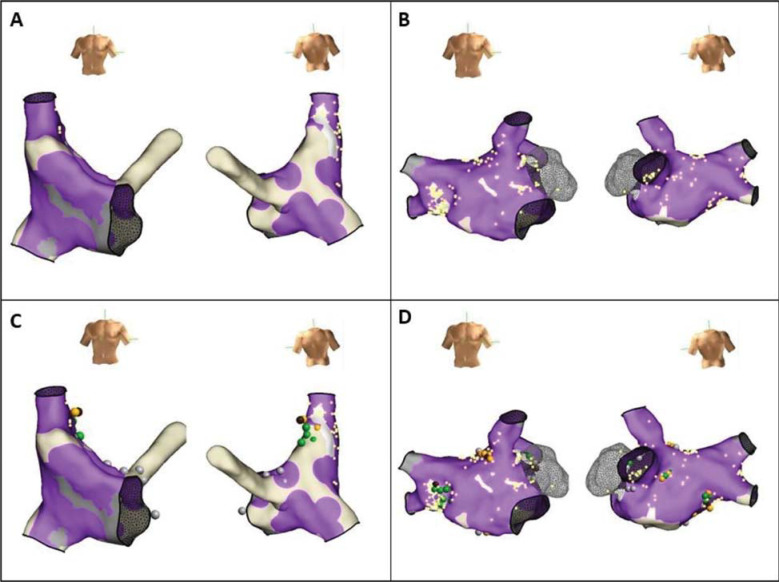
**A and B:** Fast electroanatomical map and fractionation map of the right atrium and left atria, respectively. White areas represents areas with >4 deflections suggesting ganglionated plexi localization. **C and D:** Ablation lesions in the right and left atria delivered in proximity to the right superior, posteromedial left, right inferior, left superior, Marshall tract, and left inferior ganglionated plexi.

The procedure was performed with a non-fluoroscopic approach under general anesthesia. Electroanatomic 3-dimensional mapping (EnSite™ X; Abbott, Chicago, IL, USA) and intracardiac echocardiography (ViewFlex™ Xtra; Abbott) were used. A fast anatomical map and a fractionation map of the inferior vena cava, right atrium (RA), superior vena cava (SVC) **(Figure 3A)**, and left atrium (LA) were created using the Advisor™ HD Grid mapping catheter (Abbott) **(Figure 3B)**.

A contact force ablation catheter (TactiCath™; Abbott) was used to apply lesions in the right superior ganglionated plexi (GPs) in the RA from the posteroseptal RA–SVC junction **(Figure 3C)** and additional lesions around the ostium of the coronary sinus. Ablation was performed guided by anatomy and fractionation mapping in the LA to target the right superior, posteromedial left, right inferior, left superior, Marshall tract, and left inferior GPs **(Figure 3D)**. Sites exhibiting a vagal response were ablated until the suppression of this response. Post-ablation, the heart rate was 88 bpm (44 bpm at baseline), the P–R interval was 165 ms (175 ms at baseline), and the Wenckebach point was 390 ms (490 ms at baseline).

The patient was asymptomatic during inpatient postprocedural surveillance, with no recurrence of bradyarrhythmias. The result of a new provocation “sandwich test” was negative, and post-ablation electrocardiographic changes were sustained. She was discharged for outpatient follow-up and remained asymptomatic and free from syncope during the first month after the intervention.

## Discussion

Swallowing syncope is a rare type of reflex syncope. It has been described in 0.3% of patients with recurrent syncope.^4^ Up to a third of cases with swallowing syncope may present with extracardiac pathologies mainly affecting the gastrointestinal system. As illustrated in our case, patients usually have a lengthy period of symptoms, and their manifestations are usually attributed to other explanations.^5^

Permanent pacing is used in 55% of cases with recurrent syncope and reduces the rate of recurrent syncope by 98%.^5^ CNA has emerged as an alternative therapeutic option in patients with vasovagal syncope, sinus node dysfunction, and functional vagally-mediated AV block with a reduction in recurrent episodes of syncope of 92% after 24.0 ± 11.3 months.^6^

To the best of our knowledge, this is the second reported case in the literature of treating swallow syncope with CNA^7^ and the first to be described using a zero-fluoroscopic approach. Both approaches can be considered in the treatment of patients with this condition in the future.
